# Health Tract, No. 191

**Published:** 1865

**Authors:** 


HEALTH TRACT, NO. 191.

### CURIOSITIES OF EATING.

An old beau, formerly well known in Washington City, was accustomed to eat but one meal in twenty-four hours; if, after this, he had to go to a party and take a second dinner, he ate nothing at all next day. He died at the age of seventy years.

A lady of culture, refinement, and unusual powers of observation and comparison, became a widow. Reduced from affluence to poverty, with a large family of small children dependent on her manual labor for daily food, she made a variety of experiments to ascertain what articles could be purchased for the least money, and would, at the same time, "go the farthest," by keeping her children longest from crying for something to eat. She soon discovered that when they ate buckwheat cakes and molasses, they were quiet for a longer time than after eating any other kind of food.

A distinguished Judge of the United States District Court observed that, when he took buckwheat cakes for breakfast, he could sit on the bench the whole day without being uncomfortably hungry; if the cakes were omitted, he felt obliged to take a lunch about noon. Buckwheat cakes are a universal favorite at the winter breakfast-table, and scientific investigation and analysis has shown that they abound in the heat-forming principle, hence Nature takes away our appetite for them in summer.

During the Irish famine, when many died of hunger, the poor were often found spending their last shilling for tea and tobacco and spirits. It has also been often observed in New-York, by those connected with charitable institutions, that when money was paid to the poor, they often laid out every cent in tea or coffee, instead of procuring the more substantial food, such as meal, and flour, and potatoes. On being reproved for this apparent extravagance and improvidence, the reply, in both cases, was identical; their own observation had shown them that a penny's worth of tea, or tobacco, or liquor, would keep off the sense of hunger longer than a penny's worth of any thing else. Scientific men express the idea by saying, "Tea, like alcohol, retards the metamorphosis of the tissues;" in other words, it gives fuel to the flame of life, and thus prevents it from consuming the fat and flesh of the body.

If a person gets into the habit of taking a lunch between breakfast and dinner, he will very soon find himself getting faint about the regular luncheon-time; but let him be so pressed with important engagements for several days in succession as to take nothing between meals, it will not be long before he can dispense with his lunch altogether. These things seem to show that, to a certain extent, eating often, is a mere matter of habit. Whole tribes of Indian hunters and trappers have been known to eat but once in twenty-four hours, and that at night.

